# Accurate isolation and detection of circulating tumor cells using enrichment-free multiparametric high resolution imaging

**DOI:** 10.3389/fonc.2023.1141228

**Published:** 2023-03-27

**Authors:** Dannel Yeo, Steven Kao, Ruta Gupta, Sara Wahlroos, Althea Bastian, Heidi Strauss, Vera Klemm, Prajwol Shrestha, Arturo B. Ramirez, Lillian Costandy, Ryan Huston, Brady S. Gardner, Peter Grimison, Jonathan R. Clark, John E. J. Rasko

**Affiliations:** ^1^ Li Ka Shing Cell & Gene Therapy Program, The University of Sydney, Camperdown, NSW, Australia; ^2^ Faculty of Medicine and Health, The University of Sydney, Camperdown, NSW, Australia; ^3^ Cell and Molecular Therapies, Royal Prince Alfred Hospital, Sydney Local Health District, Camperdown, NSW, Australia; ^4^ Gene and Stem Cell Therapy Program Centenary Institute, The University of Sydney, Camperdown, NSW, Australia; ^5^ Medical Oncology, Chris O’Brien Lifehouse, Camperdown, NSW, Australia; ^6^ Department of Head and Neck Surgery, Sydney Head and Neck Cancer Institute, Chris O’Brien Lifehouse, Camperdown, NSW, Australia; ^7^ NSW Health Pathology, Department of Tissue Pathology and Diagnostic Oncology, Royal Prince Alfred Hospital, Sydney Local Health District, Camperdown, NSW, Australia; ^8^ RareCyte Inc., Seattle, WA, United States; ^9^ Royal Prince Alfred Institute of Academic Surgery, Sydney Local Health District, Camperdown, NSW, Australia

**Keywords:** cancer, metastasis, liquid biopsy, biomarkers, precision medicine

## Abstract

**Introduction:**

The reliable and accurate detection of rare circulating tumor cells (CTCs) from cancer patient blood samples promises advantages in both research and clinical applications. Numerous CTC detection methods have been explored that rely on either the physical properties of CTCs such as density, size, charge, and/or their antigen expression profiles. Multiple factors can influence CTC recovery including blood processing method and time to processing. This study aimed to examine the accuracy and sensitivity of an enrichment-free method of isolating leukocytes (AccuCyte^®^ system) followed by immunofluorescence staining and high-resolution imaging (CyteFinder^®^ instrument) to detect CTCs.

**Method:**

Healthy human blood samples, spiked with cancer cells from cancer cell lines, as well as blood samples obtained from 4 subjects diagnosed with cancer (2 pancreatic, 1 thyroid, and 1 small cell lung) were processed using the AccuCyte-CyteFinder system to assess recovery rate, accuracy, and reliability over a range of processing times.

**Results:**

The AccuCyte-CyteFinder system was highly accurate (95.0%) at identifying cancer cells in spiked-in samples (in 7.5 mL of blood), even at low spiked-in numbers of 5 cells with high sensitivity (90%). The AccuCyte-CyteFinder recovery rate (90.9%) was significantly higher compared to recovery rates obtained by density gradient centrifugation (20.0%) and red blood cell lysis (52.0%). Reliable and comparable recovery was observed in spiked-in samples and in clinical blood samples processed up to 72 hours post-collection. Reviewer analysis of images from spiked-in and clinical samples resulted in high concordance (R-squared value of 0.998 and 0.984 respectively).

**Discussion:**

The AccuCyte-CyteFinder system is as an accurate, sensitive, and clinically practical method to detect and enumerate cancer cells. This system addresses some of the practical logistical challenges in incorporating CTCs as part of routine clinical care. This could facilitate the clinical use of CTCs in guiding precision, personalized medicine.

## 1 Introduction

Circulating tumor cells (CTCs) are malignant cells present in the bloodstream, originating from solid tumors. They are understood to be the ‘seeds’ of metastasis where cells shed by the primary tumor circulate through the blood, and colonize distant sites thereby forming metastatic tumors (1). The prognostic ability of CTCs has been demonstrated in various cancer types where the number of CTCs is an independent predictor of patient survival (2–5). CTCs are a potential biomarker for screening high-risk populations and for evaluating disease recurrence and metastatic spread. Early detection may provide the opportunity for initiating early treatment and thus, optimizing patient management (6–10).

CTCs analysis offers specific advantages over other biomarkers, including the ability to undertake phenotypic characterization, in real time, and in a minimally invasive manner. Because CTCs are involved in tumor progression, they provide insights into mechanisms of metastases, tumor dormancy, and through longitudinal sampling, tumor evolution and emergence of resistance. This can be undertaken by examination of their phenotypic origins using surface-antigens or by single-cell -omic analyses (11–13). The isolation of viable CTCs also allows for culturing and characterization of live cells by ex vivo culture or animal xenografts (14–16). Thus, the potential of CTCs goes beyond prognostic and diagnostic purposes and may help direct targeted, personalized treatments.

However, the detection and reliable identification of CTCs is challenging due to the low number of CTCs in the peripheral blood as well as the heterogeneity of CTCs (7, 17). Since their discovery in 1869 (18), numerous approaches have been developed to detect these rare cells in the blood, however their sensitivity, specificity and reproducibility varies widely (6, 7, 17). Many methods involve sample processing steps to enrich for CTCs are either based on their physical properties, such as size, density and charge using filters or microfluidic devices (19–21); or based on surface expression of biomarkers by immuno-magnetic capture techniques using positive (such as CellSearch^®^ for EpCAM) or negative selection (22, 23). Although there are advantages to each of these technologies, there are also limitations. Size-based capture will not detect small CTCs and a universal CTC surface marker remains elusive. Epithelial-based isolation platforms may fail to detect CTCs following their downregulation of epithelial markers as they undergo epithelial-to-mesenchymal transition (EMT) (24, 25). Furthermore, isolation of nucleated blood cells by red blood cell lysis may damage cells or remove CTCs. There is no gold standard method to determine the total number of CTCs in a patient’s blood making evaluation and comparison of these methods difficult. CellSearch, which utilizes immunomagnetic isolation of epithelial (EpCAM) CTCs, is the only FDA-approved platform for the enumeration of CTCs in breast, prostate and colon cancers. CellSearch^®^ is one of the most studied platforms but its performance varies widely (6, 7). An ideal CTC system should be sensitive enough to detect low number of CTCs with extremely high specificity to avoid false positive results, as well as be reliable for repeatability and reproducibility to overcome the potential bias of sample variability and CTC heterogeneity.

The liquid biopsy platform (RareCyte) is an enrichment-free method that involves density-based sample processing to transfer nucleated blood cells to standard glass slides (AccuCyte system) followed by immunofluorescence staining and high resolution scanning (CyteFinder instrument) to identify CTCs (26, 27). Machine learning systems also assist the reviewer by rank ordering candidate cells based on probability of being a CTC, resulting in accurate counts and reviewer concordance. AccuCyte isolates the nucleated cells using a density float-based system allowing for the float to rest at the red blood cell-plasma interface (27). This study evaluated CTC enumeration accuracy for blood samples using the AccuCyte system compared to other white blood cell isolation methods, examined different blood collection methods and processing times, and comprehensively analyzed the overall sensitivity, specificity, accuracy and precision of the AccuCyte-CyteFinder system to detect CTCs.

## 2 Methods

### 2.1 Blood sample collection

Healthy human donor blood samples were obtained from healthy volunteers after informed consent (Sydney Local Health District, Australia: 2019/ETH12590; Bloodworks Northwest, Seattle, WA: IRB#20141589). Clinical blood samples from subjects diagnosed with cancer (2 pancreatic, 1 thyroid, 1 small cell lung) were obtained after informed consent (Sydney Local Health District, Australia: 2019/ETH13813, 2021/ETH00378; St Vincent's Hospital, Australia: 2020/ETH01283). The subjects were at different disease stages: preoperative treatment naïve (thyroid); recurrence undergoing second-line therapy (pancreatic); progression after first-line therapy (small cell lung); and advanced treatment naïve (pancreatic). All methods were carried out in accordance with relevant guidelines and regulations.

Blood was collected using anticoagulant EDTA Vacutainer^®^ tubes (Becton-Dickinson) or AccuCyte^®^ blood collection tubes (RareCyte, Seattle, WA, USA) containing a proprietary preservative.

### 2.2 Source of cells for spike-in recovery samples

AsPC-1 and Capan-2 (pancreatic), and PC3 and 22Rv1 (prostate) cancer cell lines, cells known to express CK and EpCAM, were obtained from American Type Culture Collection (ATCC, Manassas, VA, USA). All cell lines were maintained in RPMI 1650 medium supplemented with 10% FBS. All cells were cultured in a 37°C incubator with 5% CO_2_.

### 2.3 Generation of spike-in recovery samples

Surrogate samples were generated using either Direct, Indirect-Supplement, or Indirect-Pool Spike-in methods. To achieve 5 cell spike-in samples, the Direct method was used where the spiked-in cell concentration was adjusted to 200 cells per microliter, then filtered to eliminate clusters. The cells were then aspirated into the CellenONE® instrument (Scienion, Berlin). Operational criteria were set to ensure deposition of single round objects one at a time directly into an AccuCyte blood collection tube containing 7.5 mL of healthy blood. For the Indirect-Supplement method, the volume of spike-in cells calculated to achieve the target cell number was added directly to the blood collection tube. This method was used for experiments requiring a set number of cells per tube only. For the Indirect-Pool method, healthy blood from multiple AccuCyte blood collection tubes were obtained from a single donor and pooled into an appropriate container. The volume of spike-in cells calculated to achieve the target cell number was added to the pool. This method was used for the linearity studies where pools corresponding to each spiked-in level were achieved by serial dilution. Because the pools contain the appropriate amount of blood collection tube preservative, they were redistributed into preservative-free evacuation tubes.

### 2.4 White blood cell isolation

**Figure 1:**
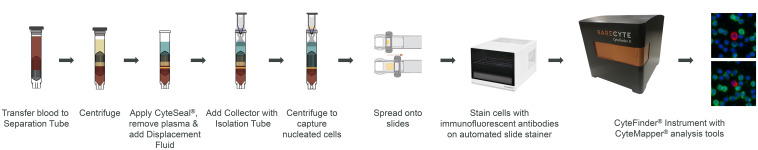
CTC enumeration workflow using the AccuCyte-CyteFinder system. AccuCyte isolation involves transferring 7.5 mL of blood to a Separation Tube and centrifuging to separate blood into the three main components: red blood cells, nucleated cell layer and plasma. A CyteSeal is applied between the red blood cell and nucleated cell layer and plasma is removed. Another centrifugation results in the capture of nucleated cells into the isolation tube. Nucleated cells are spread onto slides using the CyteSpreader device and then stained using RarePlex kits and an automated slide stainer. Slides are then scanned on the CyteFinder instrument and analyzed using the CyteMapper software to identify rare cells based on their marker expression.

Each blood sample (7.5 mL) was processed and white blood cells (nucleated cells) were isolated using either the AccuCyte system, density gradient media isolation or red blood cell (RBC) lysis methods. AccuCyte involved adding the blood to a separation tube and nucleated cells were collected into the isolation tube after two centrifugation steps (**Figure 1**) (25). Density gradient media isolation was achieved using Lymphoprep^TM^ (STEMCELL Technologies) density gradient media and harvesting the nucleated cell interface. RBC lysis involved incubating the blood sample in RBC Lysis Buffer (G-Biosciences) and centrifugation to pellet the nucleated cells.

### 2.5 Multiparametric high resolution imaging (CyteFinder instrument)

Nucleated cells were spread onto 8 Superfrost^®^ Plus slides (Thermo Scientific) using a plastic CyteSpreader^®^ device (RareCyte) and allowed to dry for 1-2 hours at room temperature. Slides were immunofluorescently stained using either RarePlex 0700-MA or 1200-VA CTC staining kits (RareCyte) on the Autostainer Link 48 (Dako–Agilent Technologies) or DISCOVERY ULTRA (Roche) stainers, respectively. The RarePlex 0700-MA CTC Assay kit includes a nuclear DAPI dye, anti-pan-cytokeratin (CK) antibody detected with CF^®^488, anti-EpCAM antibody detected with CF^®^647, and anti-CD45 antibody detected with R-phycoerythrin (PE). The RarePlex 1200-VA CTC Assay includes a nuclear Hoechst dye, anti-pan CK and anti-EpCAM antibodies detected with CF^®^647, and an anti-CD45 antibody labelled with CF^®^555. Stained slides were scanned on the CyteFinder digital immunofluorescent microscope at 10X magnification. Exposure times for the 0700-MA CTC Assay were 0.05s (DAPI), 0.025s (CK), 0.1s (EpCAM), and 0.1s (CD45); and for the 1200-VA CTC Assay: 0.1s (Hoeschst), 0.1s (CK/EpCAM), and 0.4s (CD45). Image files were analyzed by automated software (RareCyte) to define and rank score. This is performed using a proprietary machine learning algorithm that was trained on an annotated library containing thousands of images of clinical CTCs from multiple epithelial tumor types and thousands of non-CTC objects. The algorithm uses hundreds of variables which are measured on each object of interest to determine if an object on the slide is likely to be a CTC or not. Each object of interest is then assigned a score from 0 to 100 (where 100 is the highest likelihood of the object being a CTC and 0 the lowest) and ranked ordered. The software then presents images of the objects of interest in a ranked manner to a trained reviewer *via* the CyteMapper^®^ imaging analysis software (RareCyte) for final classification as CTC or not a CTC. A CTC was defined as having a nucleus, CK/EpCAM staining and no CD45 staining (27). A slide takes roughly 15mins to scan and 20mins to analyze.

### 2.6 Data analysis

Data analysis including calculations of cell recovery and concordance, was performed using GraphPad (Prism) or Excel (Microsoft). All values were expressed as means ± standard error. Differences between two means (student’s t-test) with p < 0.05 were considered significant.

## 3 Results

### 3.1 Accurate and consistent recovery at each spike-in level examined

**Figure 2:**
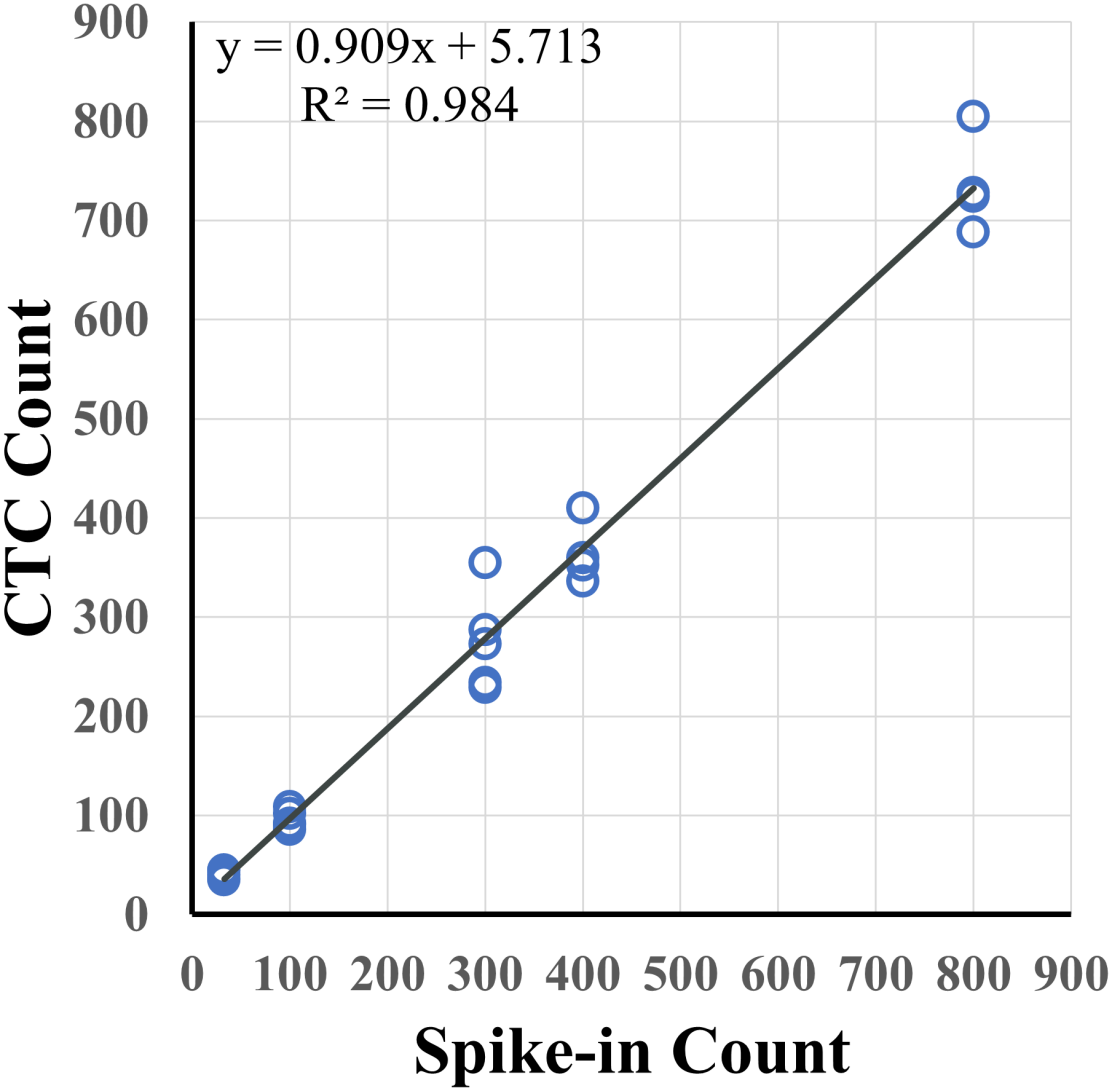
Spike-in recovery for different levels of tumor cells in 7.5 mL of healthy blood. Varying numbers of pancreatic (AsPC-1, Capan-2) and prostate (22Rv1) cancer cells were spiked into 7.5 mL of healthy blood samples with an overall recovery rate of 90.9% (R^2^ = 0.984).

Recovery rates were measured for samples containing different tumor spiked-in levels, from 30 to 800 cells, using the indirect-pool method using the AsPC-1, Capan-2 and 22Rv1 cancer cells. Recovery rate was 90.9% (R^2^ = 0.984) (**Figure 2**). To examine recovery of low cell numbers, 5 PC3 cancer cells were each spiked into 10 healthy blood samples using the direct method. For each of these 5 cancer cell spike-in samples, identification of 2 or more CTCs was required to be considered CTC-positive. Average recovery was 80% (40 out of 50 total cells, median = 90%) (**Table 1**). In parallel, 10 healthy blood samples from different donors, without any spike-in cells, showed no tumor cells (**Supplementary Data**). This resulted in a sensitivity of 90%, specificity of 100% and accuracy of 95% (**Table 2**).

**Table 1:** Recovery of rare cells using 5 spiked-in cells in healthy donor blood.

Replicate	Cells spiked-in	Cells recovered	Recovery (%)
1	5	5	100
2	5	3	60
3	5	3	60
4	5	4	80
5	5	5	100
6	5	5	100
7	5	5	100
8	5	5	100
9	5	1	20
10	5	4	80
Total	50	40	80

**Table 2:** Sensitivity, specificity and accuracy of detecting CTCs.

Replicate type	No spike-in	5 cell spike-in
Test Positive (>1 CTC)	0	9
Test Negative (≤1 CTC)	10	1
Sensitivity		0.90
Specificity		1.00
Accuracy		0.95

### 3.2 Recovery according to post-collection processing time

**Figure 3:**
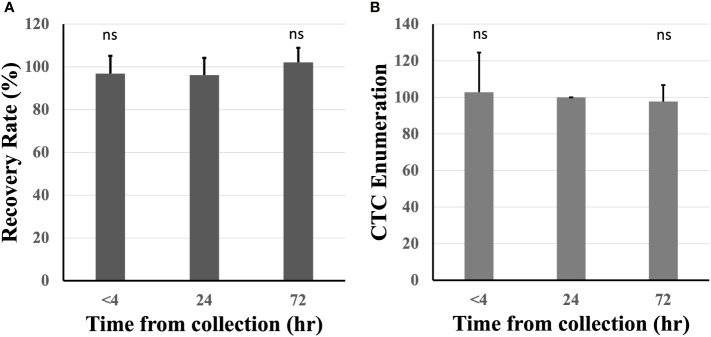
Recovery rates were maintained across different blood incubation times. Recovery rates were similar when compared at time of collection (<4 hours), 24 and 72 hours in healthy spiked-in blood **(A)**. Similar CTC enumerations were found at different incubation times in 4 subjects diagnosed with cancer (CTC counts normalized to 24 hours) **(B)**. n.s, not significant.

**Figure 4:**
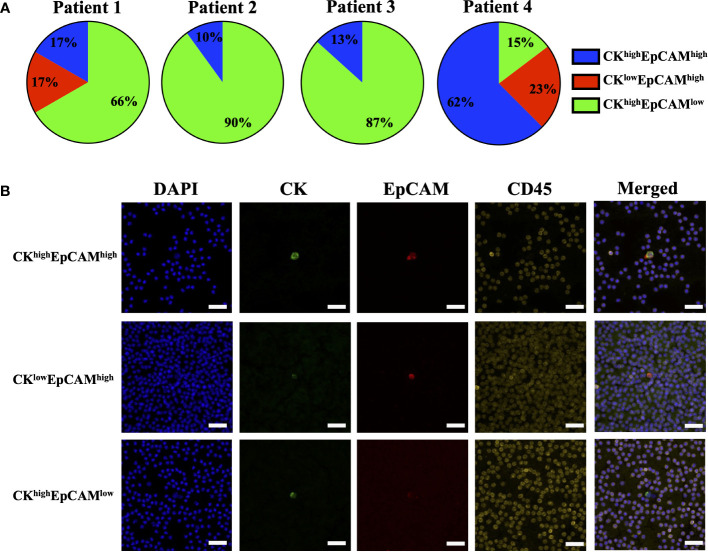
Proportions of epithelial markers in clinical sample CTCs. CTCs identified in clinical samples of patients with pancreatic cancer (Patient 1 and 2), thyroid cancer (Patient 3) and small cell lung cancer (Patient 4) had varying proportions of epithelial markers: cytokeratin (CK) and EpCAM **(A)**. Representative images are shown: CK^high^EpCAM^high^ (top), CK^low^EpCAM^high^ (middle), and CK^high^EpCAM^low^ (bottom) **(B)**. CK (green), EpCAM (red), CD45 (yellow) and nuclear DAPI (blue). Magnification = 40X; scale bar = 50 μm.

The effect of blood incubation time after collection was examined to determine CTC recovery rates using spike-in samples of AsPC-1 and 22Rv1 cancer cells using the indirect-supplement method and clinical blood samples. Spiked-in blood were processed at <4 hours (where blood was collected using an EDTA blood collection tube), 24 hours and 72 hours (AccuCyte blood collection tube). No difference in recovery rates were observed (**Figure 3A**). Clinical blood samples was collected in the same manner from 4 subjects diagnosed with cancer (2 pancreatic, 1 thyroid, 1 small cell lung) (**Table 3**). No difference in CTC detection rates was observed at the different incubation times in either spike-in or primary cancer samples (**Figure 3B**). The identified CTCs were grouped based on their epithelial marker (CK/EpCAM) fluorescence intensity (**Figures 4A, B**; **Supplementary**). Three samples exhibited high proportions of CK^high^EpCAM^low^ CTCs, one sample had high proportions of CK^high^EpCAM^high^ CTCs. CK^low^EpCAM^high^ CTCs were detected in two samples.

**Table 3:** Number of CTCs detected according to post-collection processing time.

Patient	Cancer	Status at blood collection	CTC Numbers (7.5 mL blood)		
			<4 hour	24 hours	72 hours
1	Pancreatic cancer	Recurrent cancer receiving second-line treatment	19	12	14
2	Pancreatic cancer	Advanced cancer (treatment naïve)	14	20	20
3	Thyroid cancer	Preoperative (treatment naïve)	10	15	11
4	Small cell lung cancer	Progression after first-line treatment	157	136	137

### 3.3 Recovery rate according to white blood cell isolation method

**Figure 5:**
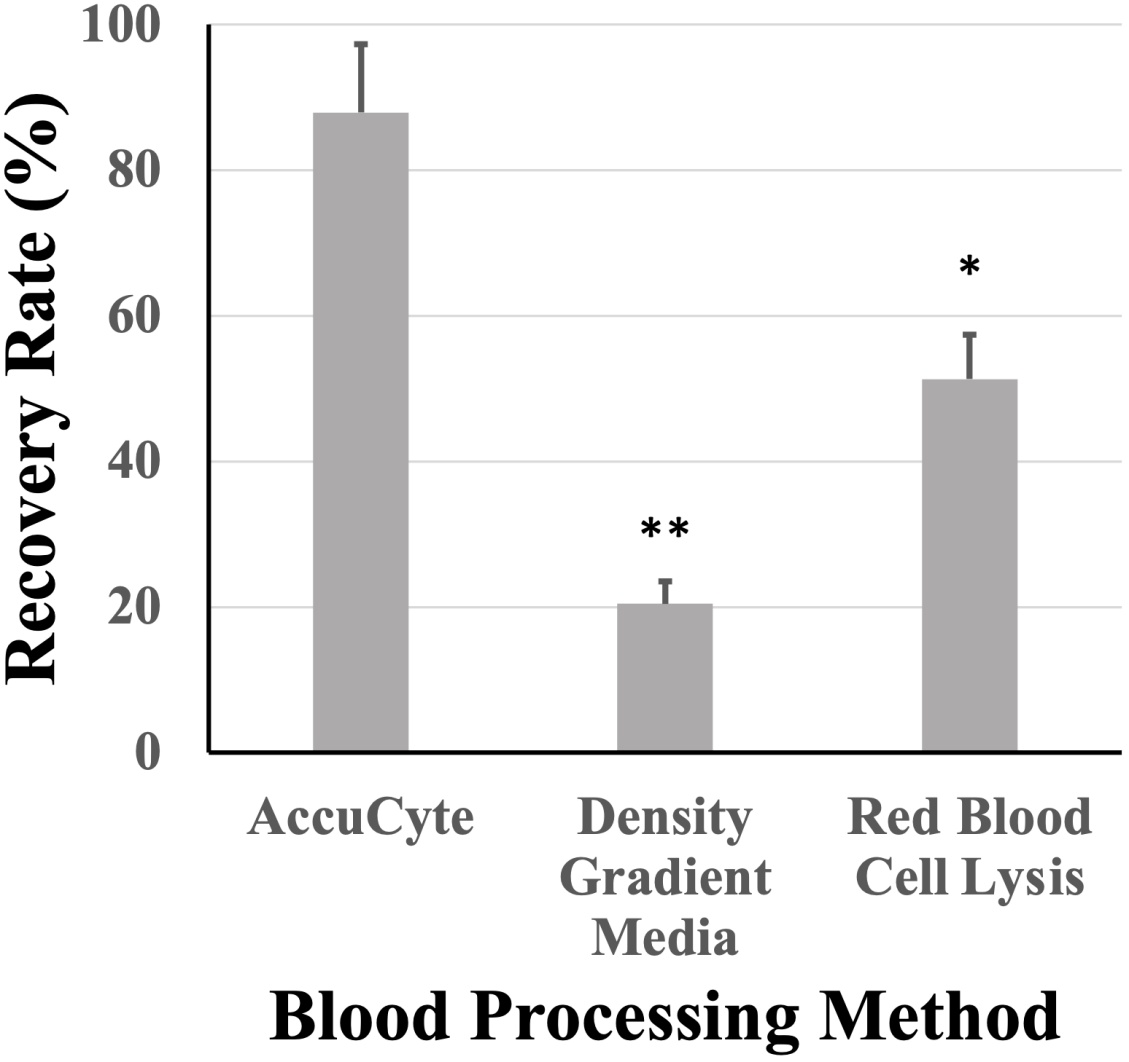
Recovery rates based on white blood cell isolation. Recovery rates from AccuCyte were superior compared to white blood cell isolation by density gradient and red blood cell lysis in spiked-in healthy blood samples. * p<0.05, ** p<0.01.

Nucleated blood cell isolation using the AccuCyte system was compared to other methods such as density gradient media isolation and RBC lysis. AccuCyte was found to be significantly superior to density gradient media isolation (p=0.004) and RBC lysis (p=0.030) (**Figure 5**).

### 3.4 Reviewer concordance

**Figure 6:**
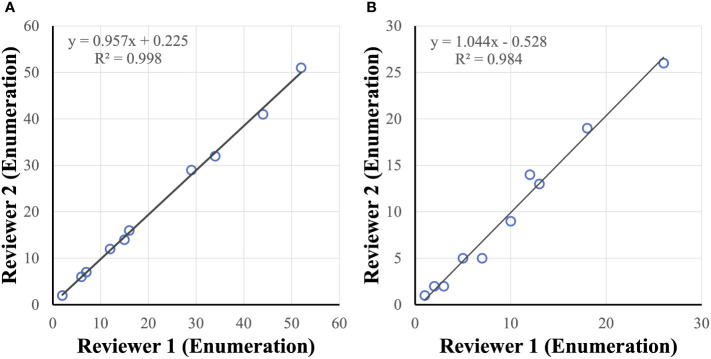
CTC reviewer concordance. Reviewers were concordant in identifying tumor cells (enumeration) in healthy spiked-in sample **(A)** and clinical sample **(B)** slides.

Cancer cell candidates are analyzed and rank-scored using machine learning algorithms followed by reviewer confirmation. To measure accuracy of cancer cell enumeration, reviewer consistency in identifying cancer cells was examined. Two reviewers, blinded to each other, were compared and found to be concordant when examining healthy spiked in samples with up to 50 cancer cells per slide (R^2^ = 0.998) and in clinical samples with up to 30 CTCs per slide (R^2^ = 0.984) (**Figure 6**). No false positive samples were identified in 6 CTC-negative clinical samples by either reviewer.

## 4 Discussion

This study demonstrates high sensitivity, specificity, accuracy, and reproducibility of the AccuCyte-CyteFinder platform for the detection of cancer cells with 90.9% recovery rate (R^2^ = 0.984; **Figure 2**). This confirms previous studies where >90% recovery rates were also observed (27–29). In addition, for the first time, consistently high recovery was observed with low number of spiked-ins cells (samples with 5 cells spiked-in) (**Table 1**), resulting in a 90% sensitivity and overall accuracy of 95%. Furthermore, we demonstrated the ability to process blood for up to 72 hours post-blood collection with no significant loss compared to day-of-draw (**Figure 3**). This could have important logistical and clinical implications as well as open opportunities for downstream analyses.

This study found that isolation of nucleated blood cells using AccuCyte was superior to other nucleated blood cell isolation methods (**Figure 5**). Isolation of nucleated blood cells by standard methods can be highly variable with a reported maximum yield of 80% as well as user-dependency (30). The AccuCyte system provided consistently high recovery of nucleated blood cells, ensuring that rare cell populations such as CTCs can be reliably identified. This study utilized staining of CK and EpCAM in different immunofluorescent channels (**Figure 4B**). While this approach differed from other published studies using the same platform that combine CK and EpCAM detection into the same channel (27–29), high recovery rates and reviewer concordance was maintained. Furthermore, recent studies have demonstrated that this platform is analytically equivalent to CellSEARCH^®^, the only FDA-approved CTC detection platform, in detecting CTCs (27, 28, 31, 32).

Several studies have utilized the AccuCyte-CyteFinder platform to detect CTCs in various cancers such as prostate, breast, and lung (9, 10, 26, 28, 29, 31–33). This study is the first to examine CTCs using this platform in thyroid cancer. Although CTCs have been found in numerous studies to be a prognostic marker (4, 6), the clinical utility could not be examined due to the small patient numbers. Clinical studies using this platform with longitudinal blood samples are required to fully elucidate the clinical utility of CTCs identified using this platform.

This study utilizes the standard CK/EpCAM biomarkers to identify CTCs. However, CTCs are known to be heterogeneous, and their expression of markers may vary (11, 12, 34). CTCs, undergoing EMT, downregulate their expression of epithelial markers (12, 35) where varying EpCAM expression was observed in the clinical samples (**Figure 4**), however, further characterization with mesenchymal markers is required. The AccuCyte-CyteFinder platform allows for up to 7 different markers allowing for additional non-epithelial biomarkers, potentially increasing CTC detection by detecting those undergoing EMT. Additional biomarkers may also be relevant to a specific tumor subtype, provide prognostic value, or to facilitate patient selection for targeted therapies. For example, PD-L1 for monitoring patients undergoing immunotherapy (10), androgen receptor splice variant 7 (ARv7) for emergence of therapeutic resistance in prostate cancer (31), and assessing biomarkers such as EGFR, HER2 or PD-L1 in esophageal cancer (36). Furthermore, the platform has a single-cell retrieval device, CellPicker™, where identified cells can be picked for single-cell molecular characterization. This includes genomic analysis such as TP53 mutations in breast cancer cells (27), APC mutations in colorectal cancer (36), targeted mutational panels in prostate (31) and lung cancer (33); and single-cell RNA sequencing as demonstrated in breast cancer (29).

In summary, our study demonstrates the sensitivity and accuracy of detecting rare cell populations such as CTCs for up to 72 hours post-blood collection, making large clinical studies operationally practical. Investigating the clinical utility of CTCs in cancer using this platform warrants larger studies. The potential downstream molecular profiling offers opportunities for clinical and research applications especially for precision/personalized patient care.

## Data availability statement

The original contributions presented in the study are included in the article/**Supplementary Material**. Further inquiries can be directed to the corresponding author.

## Ethics statement

The studies involving human participants were reviewed and approved by Bloodworks Northwest, Sydney Local Health District, St Vincent's Hospital. The patients/participants provided their written informed consent to participate in this study.

## Author contributions

DY conceived, designed, performed the analysis, and drafted the manuscript. AB, HS, VK, AR, LC, RH, BG performed the experiments. SK, RG, SW, PS, PG, JC collected the clinical samples and presentation of clinical data. SK, RG, SW, AR, LC, RH, BG, PG, JC, JR provided intellectual input. JR provided overall project conception, supervision and research governance. DY, AR, JR reviewed and edited the manuscript. All authors read and approved the final manuscript. All authors contributed to the article and approved the submitted version.
